# Synchronous diagnosis of anaplastic large cell lymphoma and multiple myeloma in a patient: A case report

**DOI:** 10.1097/MD.0000000000022931

**Published:** 2020-10-30

**Authors:** Xiaofeng Shi, Jiannong Wu, Qian Jiang, Shuo Zhang, Wanru Chen, Xianqiu Yu, Yichen Liu, Min Chen, Jie Peng, Tiantian Li, Yan Zhu, Xiaodong Xi

**Affiliations:** a Affiliated Hospital of Jiangsu University, No. 438, North Jiefang Road, Zhenjiang, Jiangsu, PR; b The Second Affiliated Hospital of Nanjing Medical University, Nanjing, Jiangsu; c Ruijin Hospital, Shanghai Jiaotong University School of Medicine, Shanghai, PR China.

**Keywords:** ALK-negative, anaplastic large cell lymphoma, co-existence, multiple myeloma

## Abstract

**Rationale:**

Synchronous development of both anaplastic large cell lymphoma (ALCL) and multiple myeloma (MM) in a patient is rare. To our knowledge, until now only one case has been reported. Treatment needs to cover both and is a challenge. Here we reported another case and discussed the diagnosis and treatment.

**Patient concerns:**

This is a 63-year old woman who presented with a mass in upper abdominal skin. Positron emission tomography/computed tomography (PET/CT) showed the high metabolism in left abdominal skin and left axillary lymph nodes. Histopathologic and immunohistochemical evaluation identified the cutaneous mass as an ALK-negative ALCL. Bone marrow smear showed increased plasma cells which expressed CD38, CD138, and cLambda concomitantly. The increased monoclonal immunoglobulin IgD λ was detected by immunofixation electrophoresis.

**Diagnoses:**

Diagnosis of both ALCL and MM was confirmed.

**Interventions:**

The patient successively received 6 cycles of B-CHOD regimen, one cycle of ID regimen, 2 cycles of DHAX regimen, one cycle of L-DA-EPOCH and autologous stem cell transplantation (ASCT). Then lenalidomide was performed as a maintenance therapy.

**Outcomes:**

Both ALCL and MM achieved complete remission.

**Lessons:**

We reported a very rare case with synchronous development of ALCL and MM, in whom a good therapeutic response to chemotherapies followed by ASCT has been observed.

## 1 Introduction

Anaplastic large cell lymphoma (ALCL), defined as a CD30 positive peripheral T-cell neoplasma, accounts for 6% to 24% T-cell lymphomas.^[1]^ Multiple myeloma (MM) is a disease defined as the B cell neoplastic proliferation of a single clone of plasma cells producing a monoclonal immunoglobulin.^[2]^ Co-existence of lymphoproliferative neoplasms of B and T-cell lineage in the same patient is rare. The development of both of ALCL and MM in the same patient one after another (metachronous) has been described in 3 cases.^[3–5]^ While the synchronous development of ALCL and MM in the same patient was reported only in one case.^[6]^ Here we reported another one in whom ALK-negative ALCL and IgD λ-type MM were diagnosed at the same time. The treatment of co-existence of ALCL and MM is challenging. Among above 4 patients, 1 was deceased,^[3]^ 2 had no records about their prognosis,^[4,5]^ and only one obtained complete remission.^[6]^ In our study this patient achieved complete remission after multiple-line chemotherapies followed by autologous stem cell transplantation (ASCT).

## 2 Case presentation

**Figure 1:**
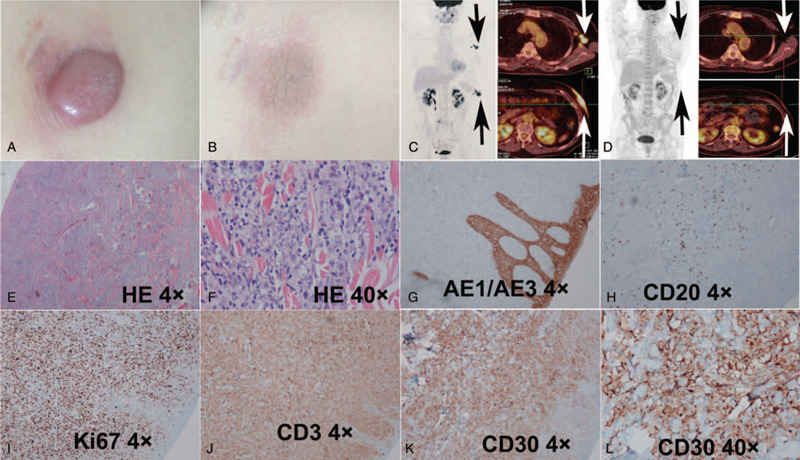
Anaplastic Large Cell Lymphoma. A and B The cutaneous lesion before (A) and after (B) chemotherapy. (C). PET/CT shows high metabolism of ^18^FDG in left abdominal skin and left axillary lymph nodes (arrows). (D). PET/CT after chemotherapy. E and F. The histopathology of HE staining reveals lymphocyte hyperplasia. G-L. The immunohistochemical results show AE1/AE3 (-), CD20 (-), Ki67 (80–90%), CD3 (+), and CD30 (+).

A 63-year old female was admitted to Hematology Department with a history of reddish papule in her abdominal skin, which developed gradually into skin nodule, and became into skin tumor in 6 months. The skin lesion exhibited no pain and no itch. Physical examination revealed the round skin mass with a diameter of 3 cm [Fig. 1 (A)] and enlarged painless left axillary lymph nodes. The histopathology of the skin biopsy revealed lymphocyte hyperplasia with increased numbers of postcapillary venules [Fig. 1 (E) and (F)]. The immunohistochemical analysis showed CD3 (+), CD5 (+), CD30 (+), MUM-1 (+), Ki67 (80–90%), CD4 (+) and ALK (-) [Fig. 1 (G-L), Table 1]. Then an anaplastic lymphoma kinase (ALK) -negative ALCL was suspected. Future fluorescence in situ hybridization (FISH) examination was ordered and the results confirmed the DUSP22 rearrangement with t (6p25) (IRF4/DUSP22) (+). The final diagnosis of ALK-negative ALCL was confirmed by Fudan University Shanghai Cancer Center through group consultation. Positron emission tomography/computed tomography (PET/CT) showed that a 7.8cm × 1.5cm × 3.3 cm mass in the abdominal skin with the maximum of standard uptake value (SUV_max_) of 14.92 and multiple left axillary lymph nodes with the SUV_max_ of 11.16 [Fig. 1 (C)]. No obvious high metabolism was found in bone. No hepatosplenomegaly was found.

**Table 1:**
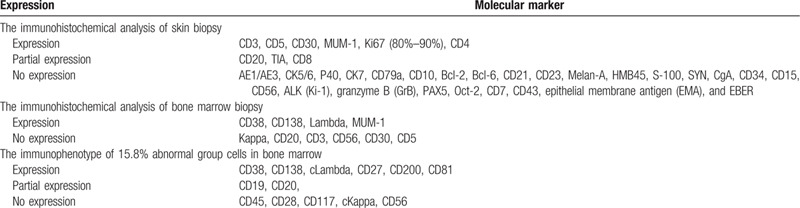
Immunohistochemical analysis and immunophenotype of skin and bone marrow.

The blood tests indicated the normal levels of whole blood counts, urea nitrogen, creatinin, serum Ca^2+^ concentration, albumin, β2-microglobulin (β2-MG), and lactate dehydrogenase (LDH). The patient had EB virus infection (DNA 3.4 × 10^3^ IU/ml) and increased erythrocyte sedimentation rate (37 mm/hour). Human immunodeficiency virus (HIV) and cytomegalovirus serology were negative. The patient had no other co-morbidities and genetic disorders, such as hypertension or diabetes, etc.

**Figure 2:**
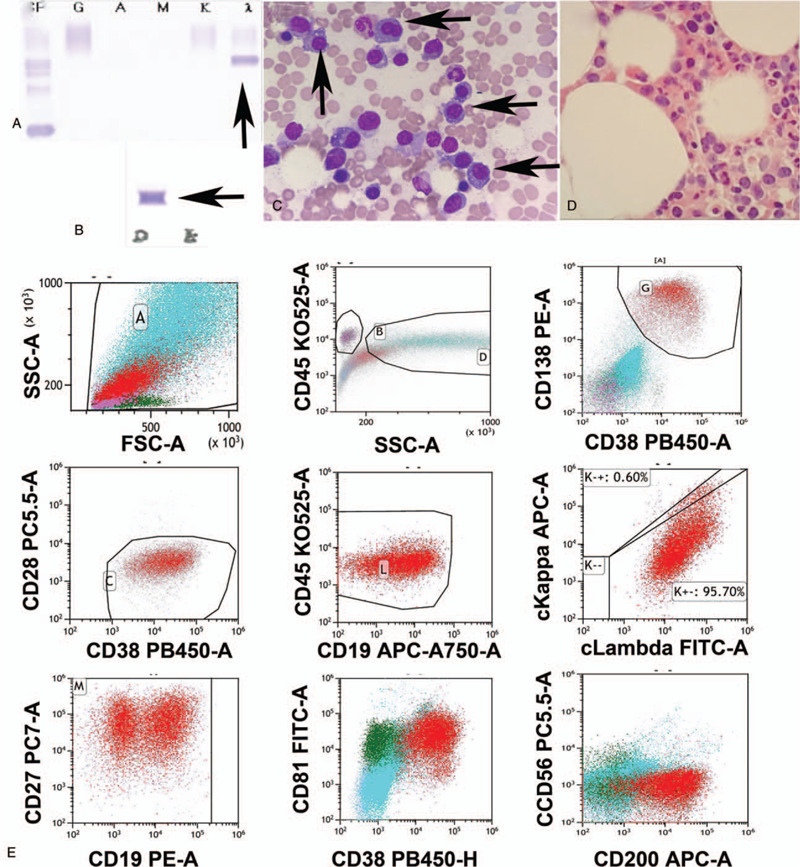
Multiple Myeloma. A and B. The IgD and λ light chains are detected by immunofixation electrophoresis. C. 26% abnormal plasma cells were found in the bone marrow smear (arrows indicate abnormal plasma cells). D. Bone marrow biopsy. E. Immunophenotype of bone marrow cells (Red group).

However, the test of serum immunoglobulin showed the IgA 0.24 g/L↓ (0.82–4.53 g/L), IgG 10.6 g/L (7.51–15.6 g/L), IgM 0.2 g/L↓ (0.46–3.04 g/L), C3 0.78 g/L↓ (0.79–1.52 g/L), C4 0.2 g/L (0.16–0.38 g/L), κ light chain 6.88 g/L (6.29–13.5 g/L), and λ light chain 17 g/L↑ (3.13–7.23 g/L). The tests of urine showed urine κ light chain 51.4 mg/L ↑ (<18.5 mg/L) and urine λ light chain 2270 ↑↑↑mg/L (<50 mg/L). The total urine λ light chain reached 2497 mg/24 hour and total κ light chain 56.54 mg/24 hour. Serum free λ light chain reached 9900 mg/L↑↑↑and κ light chain reached 8.61 mg/L. The ratio κ /λ was 0.0009↓↓↓. Serum monoclonal IgD and λ light chains were detected by immunofixation electrophoresis [Fig. 2 (A) and (B)]. Urine λ light chain band also was detected. Then the bone marrow aspiration and biopsy were performed. Twenty six percent of abnormal plasma cells were found in the bone marrow [Fig. 2 (C) and (D)]. The immunohistochemical staining of bone marrow biopsy showed CD38 (+), CD138 (+), Kappa (-), and Lambda (+) (Table 1). The flow cytometry of bone marrow showed that there were 15.8% abnormal group cells which expressed CD38, CD138, and cLambda [Fig. 2 (E), Table 1]. FISH results of del (17) (p13) (P53/CEP17), t (14;20) (q32;q12) (IGH/MAFB), t (14;16) (q32;q23) (IGH/MAF), and t (4;14) (p16;q32) (IGH/FGRF3), as well as TCR gene rearrangement, from CD138 positive selected bone marrow cells, were negative. Osteolysis was not detected in skull, ribs, long bone, and pelvis by X ray examination. The accumulation of monoclonal plasma cells in the bone marrow and monoclonal IgDλ confirmed the diagnosis of MM. Based on the SLiM symptoms, the patient belonged to an active MM.

**Figure 3:**
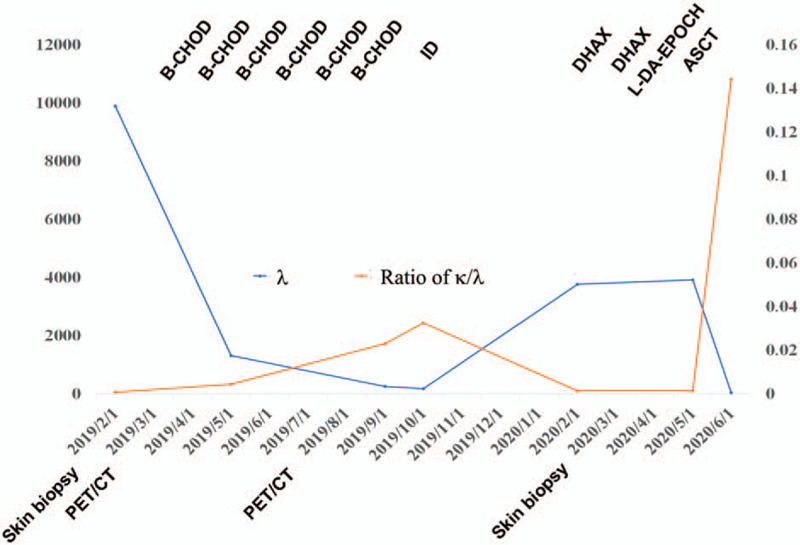
The fluctuation of λ light chain and ratio of κ/λ with the administration of treatments. B-CHOD = Bortezomiband, Cyclophosphamide, Doxorubicin, Vincristine, and Dexamethasone, ID = Ixazomib and Dexamethasone, DHAX = Oxaliplatin, Cytarabine, and Dexamethasone, L-DA-EPOCH = Lenalidomide, Etoposide, Prednisone, Vincristine, Cyclophosphamide, and Doxorubicin, ASCT = Autologous stem cell transplantation.

Then the patient received B-CHOD regimen therapy (Bortezomib i.h. day1, 8, 15, 22; Cyclophosphamide 750 mg/m^2^ i.v. day 1, Doxorubicin 50 mg/m^2^ i.v. day 1, Vincristine 1.4 mg/m^2^ i.v. day 1, and Dexamethasone 15 mg i.v. day 1–5, 8, 9, 15, 16, 22, 23). After 6 cycles of chemotherapies the cutaneous mass got complete remission and PET/CT [Fig. 1 (B) and (D)]. The immunofixation electrophoresis showed reduced IgDλ. Serum free λ light chain was 265 mg/L↑ and κ light chain was 6.1 mg/L. The ratio κ/λ was 0.023↓. Then peripheral blood stem cell mobilization was achieved following high-dose etoposide and granulocyte-colony stimulating factor, and 11.4 × 10^6^ CD34-positive cells per kilogram body weight were collected. Waiting to be transplanted, the patient started ID treatment (Ixazomib and Dexamethasone) on Oct 2019. However, due to the side effect of diarrhea she stopped treatment. On Feb 2020, red rash, which was confirmed as ALK-negative ALCL pathologically, reappeared on her posterior neck, and further blood test showed that the λ light chain reached 3775 mg/L and the ratio of κ/λ increased to 0.0016. Then the patient was managed with 2 cycles of DHAX (Oxaliplatin, Cytarabine, and Dexamethasone) and 1 cycle of L-DA-EPOCH (Lenalidomide, Etoposide, Prednisone, Vincristine, Cyclophosphamide, and Doxorubicin), but the cutaneous lesion did not ameliorate. On May 2020, ASCT was performed with BEAM (Carmustine, Etoposide, Cytarabine, and Melphalan) as the conditioning regimen. One month later the cutaneous lesion ameliorated and the λ light chain reduced to 58.1 mg/L and the ratio of κ/λ to 0.1442. Then the patient took lenalidomide orally as maintenance therapy (Fig. 3).

The ethics committee of Affiliated Hospital of Jiangsu University approved this study.

## 3 Discussion

According to the expression state of ALK protein, ALCL is classified into ALK-positive ALCL and ALK-negative ALCL. Patients with ALK-negative ALCL tend to be older, with higher LDH values, and worse performance status than ALK-positive cases.^[7]^ In ALK-negative ALCL, CD30 is expressed strongly in all tumor cells, usually in the cell membrane. A majority of ALK-negative ALCL tumor cells are positive for CD3 and negative for CD15 or PAX5. This case expressed CD30 antigen and T cell antigens (CD3, CD5), but did not express B cell antigens (CD79a, CD20) and other antigens (ALK, CD15, PAX5). Then this skin lesion fulfilled the diagnosis of ALK-negative ALCL. Meanwhile this case did not express CD56, which has been shown to be a favorable, independent prognostic factor.

In this case the enlarged lymph nodes were found during routine physical examination. So, whether the skin lesion is from an extranodal infiltration of systemic ALCL, which is more common in the ALK-negative ALCL, or from a primary cutaneous ALCL with secondary involvement of lymph nodes is unknown. Considering primary cutaneous ALCL is defined by the presence of skin involvement without evidence of extracutaneous disease for at least 6 months after presentation,^[8]^ we suspected that this case was a systemic ALCL with extranodal infiltration. The systemic ALCL behaves in an aggressive fashion, when compared to the indolent primary cutaneous ALCL. The short course and the high SUV_max_ of PET/CT in this patient indicate an aggressive feature.

DUSP22 rearrangement, which involves the DUSP22/IRF4 (Interferon Regulatory Factor 4) locus on 6p25.3, occurs in −30% of all ALK-negative ALCL and is associated with a very favorable prognosis [5-year overall survival 90%].^[9]^ The prognosis of ALK-negative ALCL with DUSP22 rearrangement is similar to that of the ALK-positive ALCL.^[10]^ The FISH result from skin biopsy showed that this patient had DUSP22 rearrangement.

MM is a B cell proliferative neoplasm.^[2]^ The criteria for diagnosis of MM include clonal bone marrow plasma cells ≥10% of biopsy-proven bony and increased monoclonal immunoglobulin. This patient fulfilled these criteria. According to the International Staging System (ISS), the patient belonged to stage I (β2-MG < 3.5 mg/L and albumin ≧35 g/L). According to the Revised ISS staging (R-ISS), she also belonged to stage I (ISS stage I and standard-risk chromosomal abnormalities by FISH and serum LDH ≦ the upper limit of normal). MM patients with Stage I have a long-term survival. Although this patient had no CRAB symptom (hyper-Calcemia, Renal insufficiency, Anemia and Bone lesions), she fulfilled the SLiM features (involved: uninvolved serum free light chain ratio ≥100). So this patient was diagnosed with an activated MM instead of a smoldering one.^[11]^ Thus, this patient needed an anti-MM therapy. In addition, lymph node enlargement caused by MM is rare, so although the axillary lymph node was not resected for biopsy, we suspected the lymph node enlargement was more associated with ALCL, than with MM. In addition, plasma cell is not ^18^F-FDG avid, and PET/CT is not very sensitive for the detection of diffuse bone marrow plasma-cell infiltration.^[12]^

Synchronous or metachronous development of lymphoproliferative neoplasms of B and T-cell lineage in the same patient is rare and still poorly understood. All the cases reported in the English language literature are described as case reports and usually associate mycosis fungoidesor and Sezary syndrome to a MM.^[13]^ To our knowledge, the coexistence of both of ALCL and MM in 1 patient one after another (metachronous) has been described in 3 cases.^[3–5]^ One case with cutaneous ALCL developed into MM 2 years later.^[3]^ Another case with 2-year history of MM developed a cutaneous ALCL in his right thigh.^[4]^ The third case developed into the cutaneous anaplastic T-cell lymphoma 16 years after the diagnosis of monoclonal gammopathy of underterminated significance (MGUS) and only 1 year after the MM diagnosis.^[5]^ While only one case, in whom ALCL and MM synchronously developed, has been reported.^[6]^ For our case, ALCL and MM developed almost at the same time (synchronous development or diagnosis). For those cases in whom ALCL succeeds to the MM, ALCL development may be secondary from the acquired cellular immunity impairment because of the fact that cutaneous ALCL is common in HIV-infected individuals^[14]^ and often found in renal and heart transplant recipients or following chemotherapy treatment.^[15]^ However, for our case, it is possible that the 2 infrequent malignancies have arisen independently of each other.

The treatment covering both 2 diseases is a challenge. For this patient, we chose regimens, including B (Bortezomiband) and CHOD (Cyclophosphamide, Doxorubicin, Vincristine, and Dexamethasone). The first-line treatment for ALCL is CHOP, in which we used dexamethasone to substitute prednisone in this patient, because dexamethasone is better than prednisone for MM. B-VAD (Bortezomib, Vincristine, Doxorubicin, and Dexamethasone) is also a primary therapy for MM patient. So, this regimen covered both of ALCL and MM. In order to facilitate stem cell mobilization in the future, lenalidomide is avoided during the initial chemotherapy. After 6 cycles of regimens, the skin lesion and lymph node enlargement obtained remission, but relapsed quickly, consistent with the fact that ALK-negative ALCL responds well to doxorubicin-containing chemotherapy, but frequently relapses. ASCT was reported in a small trial with a good response for ALCL and also is a standardized consolidation therapy for MM. One patient with co-existence of ALCL and MM achieved complete remission after ASCT.^[6]^ In this study the patient obtained a good response to ASCT. For maintenance therapy we used lenalidomide which is not only a standardized therapy for MM but also a secondary choice for ALCL. For this patient, HDAC inhibitor, chidamide, and anti-CD30 antibody, Brentuximab Vodotin, can also be considered in the future.

Synchronous diagnosis of ALCL and MM in a patient is rare. Until now only one case has been reported.^[3–6]^ Here we reported another one, in which a good therapeutic response to chemotherapy followed by ASCT has been observed.

## Author contributions

**Conceptualization:** Xiaofeng Shi, Yan Zhu.

**Formal analysis:** Xiaofeng Shi.

**Funding acquisition:** Xiaofeng Shi.

**Investigation:** Xiaofeng Shi.

**Methodology:** Yichen Liu, Min Chen, Jie Peng, Tiantian Li.

**Project administration:** Shuo Zhang, Wanru Chen, Xianqiu Yu.

**Resources:** Xiaofeng Shi, Jiannong Wu, Qian Jiang, Shuo Zhang, Wanru Chen.

**Writing – original draft:** Xiaofeng Shi.

**Writing – review & editing:** Xiaodong Xi.
